# Editorial: Equity in cancer prevention and early detection

**DOI:** 10.3389/fonc.2026.1935377

**Published:** 2026-07-30

**Authors:** Hussain Gadelkarim Ahmed, Abdelbaset Mohamed Elasbali

**Affiliations:** 1 Prof Medical Research Consultancy Center, El-Obeid, Sudan; 2 Department of Histopathology and Cytology, Faculty of Medical Laboratory Sciences (FMLS), University of Khartoum, Bukayriyah, Sudan; 3 Sulaiman Al Rajhi University, Qassim, Saudi Arabia

**Keywords:** cancer diagnosis, cancer incidence, cancer prevention, cancer screening, early detection

## Introduction

Cancer prevention and early detection interventions are among the most effective tools in reducing morbidity and mortality. Yet effectiveness is not uniform across populations. Persistent inequities in cancer incidence, stage at diagnosis, time to diagnosis, and survival reflect differences in exposure to modifiable risks and, critically, differences in access to and completion of prevention and early detection care (1). Equity must therefore be treated as a core operational requirement of cancer control systems, not as an adjunct programmatic priority.

Equity is a systems problem, not an individual deficit: Disparities in cancer outcomes are frequently discussed as if they arise primarily from patient-level factors such as health literacy, adherence, or “engagement.” While patient behaviors are important, the dominant determinants of whether prevention and screening efforts are successful often lie in health system design. While patient behaviors are important, health system design often plays a key role in determining the success of prevention and screening delivery. Often lies in health system design. Barriers such as transportation constraints, appointment scheduling friction, limited clinic availability in underserved geographies, language discordance, negative care experiences, and financial toxicity reduce uptake and completion of recommended services (2, 3). Even when eligibility criteria Even when individuals meet eligibility criteria, they may encounter care discontinuities that turn prevention and screening “coverage” into missed opportunities. Equity-focused frameworks require services to be available, realistically reachable, and dependably followed by timely diagnostic and treatment processes.

Unequal opportunity must address risk prevention: Cancer prevention includes tobacco control, vaccination (HPV and hepatitis B), and lifestyle-related risk reduction (4). However, the distribution of these interventions is often uneven due to social determinants and implementation constraints. Community-based delivery strategies, flexible hours, low-barrier scheduling, and culturally and linguistically tailored outreach can improve reach. Additionally, structural risk factors, such as neighborhood food environments, occupational exposures, and stressors linked to social inequity, shape the feasibility of lifestyle change and should be considered in prevention planning. Equity in prevention also requires active monitoring of uptake across populations. Without routine stratified reporting, disparities can remain invisible while aggregate metrics suggest success.

Screening is insufficient without diagnostic follow-up: Screening is commonly framed as the centerpiece of early detection. However, screening programs achieve their mortality benefit only when abnormal results lead to timely diagnostic resolution and appropriate treatment. Across multiple care settings, inequities emerge in the interval between screening and diagnosis (5, 6). Abnormal findings may not be communicated promptly, diagnostic appointments can be delayed, and referral processes may fail without patient navigation.

A central equity principle is that screening must be embedded in an end-to-end pathway with built-in accountability. This includes: Guaranteed result delivery through reliable patient contact methods; Time-defined follow-up standards after abnormal screening; navigation and care coordination to reduce pathway attrition; and capacity alignment so diagnostic services (imaging, pathology, and biopsy) are available when screening identifies risk (7, 8). Absent these elements, screening functions as a fragmented event rather than an equity-promoting intervention.

Reduced cost and logistical obstacles boost completion: Even insured patients can face financial toxicity from copays, deductibles, and downstream diagnostic costs. People may also need to take time off work, and their childcare needs and transportation availability can further limit their options. This situation includes time away from work, childcare needs, and transportation availability. Groups with fewer economic buffers and more precarious employment or housing disproportionately experience these burdens. These burdens are not distributed equally; they are concentrated among groups with fewer economic buffers and more precarious employment or housing situations.

Equity-oriented program design should therefore include cost transparency, assistance programs to eliminate out-of-pocket barriers where possible, scheduling models that accommodate working families, and community-based delivery options (including mobile units or local partner sites). Language access and disability accommodations must be treated as part of quality, not as optional add-ons.

Measurement for equity: Beyond participation to outcomes: Equity requires measurement that captures both access and effectiveness (9, 10). Participation rates alone can mask inequities if follow-up is incomplete or delayed. Recommended equity metrics should include: Screening completion by sociodemographic and geographic stratifiers; Time from an abnormal result to diagnostic resolution; Follow-up completion after an abnormal screening and the receipt of recommended diagnostic workups. “Stage at diagnosis refers to the proportion of patients who present with advanced-stage disease. In advanced-stage presentation, patient-reported experiences, including communication quality, respect, and trust, as well as incidence and mortality trends where feasible, should be considered. Data systems should support continuous quality improvement rather than one-time disparity reporting. Importantly, equity metrics must be reviewed by program leaders with resources tied to performance.

Policy and practice implications: Equitable cancer prevention and early detection require coordinated actions across clinical care, public health, and health system operations (11). Evidence-informed strategies include expanding community-based delivery, implementing patient navigation, strengthening care coordination, improving language and disability access, and ensuring diagnostic capacity after abnormal screening. Policy should incentivize end-to-end pathway performance rather than short-term screening volume.

In conclusion, equity in cancer prevention and early detection is achieved when systems ensure that prevention tools and early diagnostic pathways reliably reach and serve populations who have historically been disadvantaged. This requires shifting from a model of eligibility and availability to one of accountable delivery, where screening is paired with timely follow-up, barriers are actively reduced, and outcomes are monitored across groups. Equity is not merely ethical; it is foundational to the effectiveness of cancer control. To reduce the cancer burden, we must prevent delays, close gaps in follow-through, and build pathways that function in the realities of patients’ lives.

## References

[1] Ayenigbara IO . Risk-reducing measures for cancer prevention. Korean J Fam Med. (2023) 44:76–86. doi: 10.4082/kjfm.22.0167 36966737 PMC10040267

[2] Al Shamsi H Almutairi AG Al Mashrafi S Al Kalbani T . Implications of language barriers for healthcare: A systematic review. Oman Med J. (2020) 35:e122. doi: 10.5001/omj.2020.40 32411417 PMC7201401

[3] Ngondwe P Tefera GM . Barriers and facilitators of access to healthcare among immigrants with disabilities: A qualitative meta-synthesis. Healthcare (Basel). (2025) 13:313. doi: 10.3390/healthcare13030313 39942501 PMC11816456

[4] Anand P Kunnumakkara AB Sundaram C Harikumar KB Tharakan ST Lai OS . Cancer is a preventable disease that requires major lifestyle changes. Pharm Res. (2008) 25:2097–116. doi: 10.1007/s11095-008-9661-9 18626751 PMC2515569

[5] Loud JT Murphy J . Cancer screening and early detection in the 21st century. Semin Oncol Nurs. (2017) 33:121–8. doi: 10.1016/j.soncn.2017.02.002 28343835 PMC5467686

[6] Patole VP Shinde NV Chandak SM Satalkar AT . Cancer research in the 21st century: Recent advances and future perspectives. Zhongguo Ying Yong Sheng Li Xue Za Zhi. (2025) 41:e20250018. doi: 10.62958/j.cjap.2025.018 40628691

[7] Coronado GD Gautom P Schneider JL Edelmann AC Coury J Doescher M . Multilevel challenges and adaptations to patient navigation programs for colorectal cancer screening and follow-up: Findings from the Accelerating Colorectal Cancer Screening and follow-up through Implementation Science consortium. Cancer. (2025) 131:e70121. doi: 10.1002/cncr.70121 41060871 PMC12507143

[8] Raich PC Whitley EM Thorland W Valverde P Fairclough D Denver Patient Navigation Research Program . Patient navigation improves cancer diagnostic resolution: An individually randomized clinical trial in an underserved population. Cancer Epidemiol Biomarkers Prev. (2012) 21:1629–38. doi: 10.1158/1055-9965.EPI-12-0513 23045537 PMC4053249

[9] Roth H De Marchis E Kopaskie K Restall A Fichtenberg C Ray S . Measure what matters: A survey-based examination of health equity tracking and measurement practices across healthcare systems in the United States. PloS One. (2025) 20:e0323381. doi: 10.1371/journal.pone.0323381 40397918 PMC12094744

[10] Heidenreich P Sandhu A . Pursuing equity in performance measurement. Circulation. (2023) 147:1134–6. doi: 10.1161/CIRCULATIONAHA.123.064123 37036909 PMC10088056

[11] Miller SJ Sly JR Jandorf L Minardi F Beyrouty MW Taioli E . Cancer prevention in a postpandemic world: A one-stop-shop approach. Am J Prev Med. (2022) 63:146–8. doi: 10.1016/j.amepre.2022.01.032 35410772 PMC8993046

